# *RET* Germline Mutations Identified by Exome Sequencing in a Chinese Multiple Endocrine Neoplasia Type 2A/Familial Medullary Thyroid Carcinoma Family

**DOI:** 10.1371/journal.pone.0020353

**Published:** 2011-05-31

**Authors:** Xiao-Ping Qi, Ju-Ming Ma, Zhen-Fang Du, Rong-Biao Ying, Jun Fei, Hang-Yang Jin, Jian-Shan Han, Jin-Quan Wang, Xiao-Ling Chen, Chun-Yue Chen, Wen-Ting Liu, Jia-Jun Lu, Jian-Guo Zhang, Xian-Ning Zhang

**Affiliations:** 1 Department of Urologic Surgery and Department of Pathology, The 117th PLA Hospital, Hangzhou, Zhejiang, China; 2 Department of Biochemistry and Genetics, Zhejiang University-Adinovo Center for Genetic and Genomic Medicine, National Education Base for Basic Medical Sciences, School of Medicine, Zhejiang University, Hangzhou, Zhejiang, China; 3 Department of Oncologic Surgery, Tumor Hospital of Taizhou, Wenling, Zhejiang, China; 4 BGI-Shenzhen, Beishan Industrial Zone, Yantian District, Shenzhen, Guangdong, China; Ohio State University Medical Center, United States of America

## Abstract

**Background:**

Whole exome sequencing provides a labor-saving and direct means of genetic diagnosis of hereditary disorders in which the pathogenic gene harbors a large cohort of exons. We set out to demonstrate a suitable example of genetic diagnosis of MEN 2A/FMTC (multiple endocrine neoplasia type 2/familial medullary thyroid carcinoma) using this approach.

**Methodology/Principal Findings:**

We sequenced the whole exome of six individuals from a large Chinese MEN2A/FMTC pedigree to identify the variants of the *RET* (REarranged during Transfection) protooncogene and followed this by validation. Then prophylactic or surgical thyroidectomy with modified or level VI lymph node dissection and adrenalectomy were performed for the carriers. The cases were closely followed up. Massively parallel sequencing revealed four missense mutations of *RET*. We unexpectedly discovered that the proband's daughter with MEN 2A-related MTC presented a novel p.C634Y/V292M/R67H/R982C compound mutation, due to the involvement of p.C634Y in the proband with MEN 2A and p.V292M/R67H/R982C in the proband's husband with FMTC. In the maternal origin, p.C634Y caused bilateral MTC in all 5 cases and bilateral pheochromocytoma in 2 of the 5; the earliest onset age was 28 years. In the paternal origin, one of the six p.V292M/R67H/R982C carriers presented bilateral MTC (70 years old), one only had bilateral C-cell hyperplasia (44 years), two had bilateral multi-nodules (46 and 48 years) and two showed no abnormality (22 and 19 years).

**Conclusions/Significance:**

The results confirmed the successful clinical utility of whole exome sequencing, and our data suggested that the p.C634Y/V292M/R67H/R982C mutation of *RET* exhibited a more aggressive clinical phenotype than p.C634Y or p.V292M/R67H/R982C, while p.V292M/R67H/R982C presented a relatively milder pathogenicity of MTC and likely predisposed to FMTC.

## Introduction

The REarranged during Transfection (*RET*) protooncogene containing 21 exons is mapped on chromosome 10q11.2 and encodes a tyrosine kinase receptor that plays a crucial role in regulating cell proliferation, migration, differentiation, and survival through embryogenesis [1], [2]. Germline mutations of *RET* are found in multiple endocrine neoplasia type 2 (MEN 2) and Hirschsprung disease (HSCR) in an autosomal dominant pattern [3], [4]. MEN 2 is subtyped into MEN 2A, MEN 2B, and familial medullary thyroid carcinoma (FMTC) based on the different tissues involved [5]. MEN 2A, the most common clinical subtype, is characterized by the MTC triad in nearly 95%, pheochromocytoma (PHEO) in 50–57%, and hyperparathyroidism (HPT) in 15–30% of cases [5]–[7]. MEN 2B is similar to MEN 2A except that HPT is rare, and characteristic developmental abnormalities, such as marfanoid habitus and ganglioneuromatosis of the intestinal tract, are present [5]. FMTC is defined by the sole presence of MTC as the disease phenotype [5]. MEN 2 and HSCR are usually distinct but occasional families have been reported with both diseases [4]. In HSCR, *RET* mutations are dispersed throughout the gene with poor genotype-phenotype correlation [4], while all the MEN 2 mutations target within exons 5, 8, 10, 11, and 13 to 16 with strong genotype-phenotype correlations [5]–[7]. More than 95% of MEN2A and FMTC patients have a *RET* mutation typically occurring at one of the five cysteine residues (codons 609, 611, 618, and 620 within exon 10, and codon 634 within exon 11). Biochemical and biological analyses revealed that these cysteine mutations induce disulfide-linked dimerization of the RET protein, leading to constitutive activation of its intrinsic tyrosine kinase activity [8]. Other mutations within exons 8, 10, 11, and 13 to 16 appear to account for a small percentage of FMTC families [7], [9]. MEN 2B is mainly associated with mutation of codon 918 within exon 16 [5]. A second mutation at codon 883 within exon 15 and tandem *RET* mutations of codons 805, 806, and 904 in *cis* configuration with the p.V804M mutation have also been reported in individuals with MEN 2B [4], [8], [10], [11]. Recently, Castellone *et al* found a p.V292M mutation within exon 5 in an Italian man with MTC and PHEO and suggested that the mutant had low-grade transforming potential [12]. Emerging reports of compound *RET* mutations appear to show modulation of the degree of aggressiveness and the development of unusual features of MTC [13]. p.R982C, a rare polymorphism within exon 18 with a frequency of about 2%, was first reported in HSCR [14]. p.R67H combined with p.R982C was reported to be associated with congenital central hypoventilation syndrome [15].

Protein-coding genes constitute only approximately 1% of the human genome but harbor nearly 85% of the disease-causing mutations at individual Mendelian loci [16]–[18]. Therefore, efficient strategies for selectively sequencing complete coding regions can serve as a genome-wide scan for the pathogenic gene [17]–[19]. Whole exome capture and massively parallel DNA sequencing has provided a labor-saving, reproducible and robust method for disease gene discovery and clinical diagnosis [18], [20]. Here, we used whole exome sequencing (WES), coupling the Agilent whole exome capture system to the Illumina HiSeq 2000 DNA sequencing platform, to identify a Chinese female with MEN 2A-related MTC carrying the p.C634Y/V292M/R67H/R982C mutation of *RET*, in whom p.C634Y was maternal with 5 carriers with MEN2A, and p.V292M/R67H/R982C was paternal with 6 carriers.

## Methods

### Subjects

**Figure 1:**
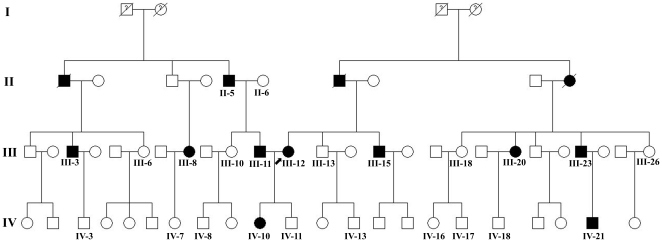
Pedigree of the family investigated (numbers indicate available).

A 22-year-old woman with MEN 2A-related MTC and her brother, and 10 paternal and 12 maternal individuals from a four-generation southern Chinese family (Fig. 1), and 100 unrelated healthy matched controls were studied.

This study was conducted in conformity with the Helsinki Declaration and approved by the Ethics Committee of the 117^th^ PLA Hospital. Written informed consent was given by all subjects.

### Clinical Investigation

Clinical evaluation of MEN 2A/FMTC was performed according to the published criteria [6], [7], [21]. The following parameters were studied: clinical and diagnostic data (age, gender and clinical features), biochemical markers (serum calcitonin (Ct), carcinoembryonic antigen (CEA), parathyroid hormone, catecholamines), Doppler ultrasound (US) and computerized tomography (CT) scans. Prophylactic or surgical thyroidectomy with neck dissection and adrenalectomy was performed after confirmation of *RET* mutations for the diagnosis. Then follow-up was carried out.

### Exome Capture and Massively Parallel DNA Sequencing

The peripheral blood genomic DNA samples from each of six family members (II-5, III-10, III-11, III-12, IV-10 and IV-11) were randomly fragmented by Covaris to provide DNA fragments with a base pair peak at 150 to 200 bp, then adapters were ligated to both ends of the resulting fragments. The adapter-ligated templates were purified by Agencourt AMPure SPRI beads, and fragments with an insert size of ∼200 bp were excised. Extracted DNA was amplified by ligation-mediated PCR (LM-PCR), purified, and hybridized to the SureSelect Biotinylated RNA Library for enrichment; hybridized fragments were bound to strepavidin beads whereas non-hybridized fragments were washed out. Captured LM-PCR products were subjected to an Agilent 2100 Bioanalyzer to estimate the magnitude of enrichment. Each captured library was then loaded onto the Hiseq2000 platform, and we performed GA sequencing for each captured library independently to ensure each sample had at least 50-fold coverage. Raw image files were processed by Illumina Pipeline v1.6 for base-calling with default parameters and the sequences of each individual were generated as 90 bp paired-end reads.

SOAPaligner (soap2.21) was used to align the clean reads to the human reference genome. Based on the SOAP alignment results, the software SOAPsnp was used to assemble the consensus sequence and call genotypes in target regions [22]. We filtered the SOAPsnp results as follows: base quality more than 20, depth between 4 and 200, estimate copy number equal to or less than 2 and the distance between two SNPs no less than 5. Detailed information of each parameter for the two algorithms is available at http://soap.genomics.org.cn/.

Data from the dbSNP129 and 1000 Genome Project (20100208 release) were downloaded from the NCBI FTP site (http://www.ncbi.nlm.nih.gov/Ftp/). Target regions were from the captured regions of the Agilent Exome Array (http://www.nimblegen.com/downloads/annotation/seqcap_exome/index.html). The exome variation set of eight HapMap samples was obtained from “Supplementary data 2” of 12 exomes in Ng *et al* [20].

### *RET* Mutation Confirmation

To further confirm the *RET* substitution mutations and the pattern of the compound mutation in the proband's daughter, we isolated DNA and total RNA from peripheral blood and tumor tissues (MTC from III-11 and III-20, PHEO from III-12) respectively, and performed polymerase chain reaction (PCR) amplification of all *RET* exons followed by Sanger sequencing, restriction endonuclease digestion, reverse transcription PCR (RT-PCR)/sequencing, and haplotype analysis of four *RET*-flanking microsatellite markers (D10S197, D10S196, D10S1652 and D10S537).

All new data have been deposited in GenBank.

## Results

### Clinical Features and Phenotypic Data

The proband (III-12) was a 43-year-old woman who had been diagnosed as having MTC in 1995 with a 4-year history of a palpable neck mass in the right thyroid. Biochemical examination disclosed increased CEA (17.7 ng/mL, normal <5 ng/mL) and Ct (408.32 ng/L, normal <100 ng/L), while US scanning disclosed multi-centric hypoechoic nodules with calcifications in both thyroid lobes. Then a total bilateral thyroidectomy with modified neck dissection was performed and histopathological examination showed bilateral multi-centric MTC with lymph node metastases (T2N1aM0, Table 1). Since surgery, she has been receiving L-T4 substitution therapy. Her father died of thyroid cancer in 1992. In 2001, the proband's 13-year-old daughter (IV-10) was diagnosed as having MTC with a 1-year history (first diagnosed as multi-nodular goiter rather than MTC). Surveillance found that the palpable neck mass in the left thyroid lobe increased in size and reached a maximum diameter of 3.9 cm. Ct was elevated to 343.92 ng/L and CEA was 14.7 ng/mL. US and CT scanning disclosed multi-centric nodules with calcifications in both lobes. Then, IV-10 received a total thyroidectomy with modified bilateral neck dissection. Histopathology revealed multi-centric MTC with lymph node metastases (T2N1aM0, Table 1). In 2005, the proband's 45-year-old male cousin (III-23) presented with headache, paroxysmal hypertension, dizzy spells, and excess serum catecholamine (Tables 1 and 2). US and CT scanning confirmed bilateral masses in the adrenal glands. After treatment with α-blockers, he underwent a total bilateral laparoscopic adrenalectomy with CO_2_ pneumoperitoneum; bilateral PHEO was confirmed by histopathological examination. In 2006, the proband's 52-year-old female cousin (III-20) underwent a total thyroidectomy with modified bilateral neck dissection based on the evidence of elevated Ct and CEA and multi-centric nodules with calcifications in both thyroid lobes but without adrenal abnormalities. In 2010, some previously hesitant members of the family (III-13, III-15, III-18, III-26, IV-11, IV-13, IV-16, IV-17, IV-18 and IV-21) agreed to participate in biochemical testing, image examinations and *RET* screening (Tables 1 and 2). All had normal Ct, CEA and US images except III-15, III-23, IV-21 and III-12 with p.C634Y. Then a total thyroidectomy with modified bilateral (III-15 and III-23) or bilateral level VI lymph node dissection (IV-21) and a total bilateral laparoscopic adrenalectomy with CO_2_ pneumoperitoneum (III-12) were performed. Histopathological examination revealed bilateral PHEO in III-12 and bilateral multi-focal MTC with lymph node metastases in III-15, III-20 and III-23, while IV-21 showed no evidence of lymph node metastases. All six patients (III-12, III-15, III-20, III-23, IV-10 and IV-21) were followed up. Biochemical and imaging examination did not reveal any evidence of recurrence of MTC or PHEO, and showed no HPT during this period except for III-12. Biochemical examination of III-12 showed Ct was 1360 ng/L and CEA was 13.2 ng/mL. US-guided fine needle aspiration and cytology was suggestive of MTC with lymph node metastases.

**Table 1:** Clinical presentations of patients with MTC and *RET* mutations.

Individual	Sex	Age	Mutation and origin	Pre-op Ct	US results	Histology	ADM (years)	LN+/resected^a^	pTNM
IV-10	F	22	p.C634Y/V292M/R67H/R982C, Pat and Mat	343.92	Negative; TT	Bi-MTC	13 (onset)	5/51	T2N1aM0 (stage III)
II-5	M	70	p.V292M/R67H/R982C, Pat	76.35	Negative; TT	Bi-MTC	70	0/15	T1N0M0 (stage I)
III-3	M	48	p.V292M/R67H/R982C, Pat	49.86	Negative; AS	No spec	48	No spec	No spec
III-8	F	46	p.V292M/R67H/R982C, Pat	43.72	Negative; AS	No spec	46	No spec	No spec
III-11	M	44	p.V292M/R67H/R982C, Pat	58.66	Negative; TT	Bi-CCH	44	-	-
IV-3	M	22	p.V292M/R67H/R982C, Pat	39.34	Normal	-	-	-	-
IV-7	F	19	p.V292M/R67H/R982C, Pat	59.21	Normal	-	-	-	-
III-12*	F	43	p.C634Y, Mat	408.32	Negative; TT	Bi-MTC	28 (onset)	3/40	T2N1aM0 (stage III)
III-15	M	41	p.C634Y, Mat	104.35	Negative; TT	Bi-MTC	39	2/22	T1N1aM0 (stage III)
III-20	F	56	p.C634Y, Mat	260.49	Negative; TT	Bi-MTC	52 (onset)	3/23	T1N1aM0 (stage III)
III-23	M	50	p.C634Y, Mat	163.23	Negative; TT	Bi-MTC	50 (onset)	2/26	T2N1aM0 (stage III)
IV-21	M	25	p.C634Y, Mat	34.70	Negative; TT	Bi-MTC	24	0/15	T1N0M0 (stage I)

*proband; F, female; M, male; Pat, paternal family; Mat, maternal family; Pre-op Ct, pre-operative calcitonin (ng/L); US, ultrasound; AS, awaiting surgery; ADM, age at diagnosis of MTC; pTNM, tumour stage; TT, total thyroidectomy; Bi-, Bilateral; No spec, no specimen.

a LN+ includes positive lymph nodes proven on histopathology; resected includes lymph node resected.

**Table 2:** Clinical presentations of PHEO with *RET* mutations.

Individual	Sex	Age at onset(yr)	Mutation and origin	Catecholamine serum level (ng/L) (Normal: DA<100; E<600; NE<100)	Tumor size	AT	Histology
III-12	F	43	p.C634Y, Mat	Normal	L: 2.5×2.0×2.0 cm; R: 1.5×1.2×1.0 cm	Yes	Bi-PHEO
III-23	M	45	p.C634Y, Mat	Elevated (DA: 278.13; E: 925; NE: 6670.7)	L: 4.3×3.6×2.8 cm; 1.2×0.8×0.8 cm; R: 4.5×2.2×1.6 cm	Yes	Bi-PHEO

F, female; M, male; Mat, maternal family; DA, dopamine; E, epinephrine; NE, norepinephrine; L, left; R, right; AT, adrenalectomy; Bi-, Bilateral.

The proband's husband (III-11) and his family members have not felt any special abnormality to date. As p.V292M/R67H/R982C and p.C634Y/V292M/R67H/R982C mutations of *RET* were found by WES in him and his daughter (IV-10) respectively in 2010, neck US screening was performed on all 6 carriers and the results disclosed multi-focal hypoechoic nodules (range 0.4–0.8 cm) in the upper third of left/right or both thyroid lobes in II-5, III-3, III-8 and III-11 but not in IV-3 and IV-7. Ct was normal while the pentagastrin-stimulated Ct was slightly elevated in II-5 and III-11. In 2010, a total thyroidectomy with modified bilateral level VI lymph node dissection was performed in II-5, then histopathological examination revealed bilateral MTC without lymph node metastases (T1N0M0, Table 1). III-11 underwent a prophylactic total thyroidectomy in 2010 at the age of 44, and histopathology revealed bilateral multi-focal C-cell hyperplasia (CCH) without evidence of MTC. III-3 and III-8 await thyroidectomy, while IV-3 and IV-7 are being followed up (Table 1). None of the 6 carriers showed abnormalities in the parathyroid or adrenal glands.

We excluded the possibility of HSCR in this family based on the fact that none of the affected individuals presented evidence of HCSR involving severe constipation, vomiting, abdominal distension, or enterocolitis. Meanwhile, cutaneous lichen amyloidosis was also excluded because there was no evidence of pruritic lichenoid skin lesion located on the upper back.

### Exome Sequencing Identified Four *RET* Missense Variants

We generated an average of approximately 4.0 billion bases of sequence per affected individual as paired-end, 88-bp reads, and the fraction of effective bases on target was about 64% with a minimum 53-fold of average sequencing depth on target. At this depth of coverage, more than 99% of the targeted bases were sufficiently covered to pass our thresholds for variant calling (Table 3). We focused on nonsynonymous (NS) variants, splice acceptor, and donor site mutations (SS), anticipating that synonymous variants would be far less likely to be pathogenic. Filtering against public SNP databases, the 1000 Genome Project, eight HapMap exomes, the in-house exome database BGI provided and two normal individuals from the family, 30 genes harboring 95 different NS/SS were shared by the four patients. We just focused on the *RET* gene and found four recurrent missense substitutions: c.G200A (p.R67H), c.G874A (p.V292M), c.G1901A (p.C634Y) and c.C2944T (p.R982C) (Sequence Read Archive accession number: SRA029363.2). Analysis to explain or eliminate the pathogenicity of the other 29 genes (including 90 NS/SS) in MEN2A is ongoing and further data will be reported elsewhere.

**Table 3:** Overview of whole exome sequencing data production.

Sample	II-5	III-10	III-11	III-12	IV-10	IV-11
Initial bases on target:	37,640,396	37,563,132	37,640,396	37,563,132	37,563,132	37,640,396
Initial bases near target:	57,236,802	57,118,957	57,236,802	57,118,957	57,118,957	57,236,802
Initial bases on or near target:	94,877,198	94,682,089	94,877,198	94,682,089	94,682,089	94,877,198
Total effective reads:	41,670,096	42,750,582	41,649,379	40,395,197	48,247,010	42,912,533
Total effective yield (Mb):	3,660.53	3,756.66	3,645.29	3,567.62	4,238.35	3,771.98
Average read length (bp):	87.85	87.87	87.52	88.32	87.85	87.90
Effective sequence on target (Mb):	2,350.98	2,380.88	2,328.08	2,277.75	2,779.49	2,405.11
Effective sequence near target (Mb):	531.72	572.00	495.98	530.61	599.20	544.23
Effective sequence on or near target (Mb):	2,882.70	2,952.88	2,824.06	2,808.36	3,378.69	2,949.34
Fraction of effective bases on target:	64.20%	63.40%	63.90%	63.80%	65.60%	63.80%
Fraction of effective bases on or near target:	78.80%	78.60%	77.50%	78.70%	79.70%	78.20%
**Average sequencing depth on target:**	**62.46**	**63.38**	**61.85**	**60.64**	**74.00**	**63.90**
Average sequencing depth near target:	9.29	10.01	8.67	9.29	10.49	9.51
Mismatch rate in target region:	0.57%	0.57%	0.56%	0.56%	0.59%	0.56%
Mismatch rate in all effective sequence:	0.59%	0.59%	0.58%	0.57%	0.61%	0.58%
Base covered on target:	37,265,593	37,219,081	37,283,514	37,159,448	37,260,142	37,287,524
**Coverage of target region:**	**99.00%**	**99.10%**	**99.10%**	**98.90%**	**99.20%**	**99.10%**
Base covered near target:	45,466,110	46,437,695	44,284,058	45,892,786	45,603,313	46,116,042
Coverage of flanking region:	79.40%	81.30%	77.40%	80.30%	79.80%	80.60%
Fraction of target covered with at least 20×	80.60%	81.70%	80.30%	80.20%	84.50%	81.40%
Fraction of target covered with at least 10×	91.30%	91.90%	91.20%	91.00%	93.10%	91.70%
Fraction of target covered with at least 4×	96.70%	96.90%	96.70%	96.50%	97.30%	96.90%
Fraction of flanking region covered with at least 20×	14.00%	15.30%	12.90%	13.90%	16.00%	14.40%
Fraction of flanking region covered with at least 10×	27.90%	29.90%	26.00%	28.10%	29.90%	28.60%
Fraction of flanking region covered with at least 4×	49.70%	52.20%	47.10%	50.40%	51.20%	50.90%

### Validation of the *RET* Germline Mutation

**Figure 2:**
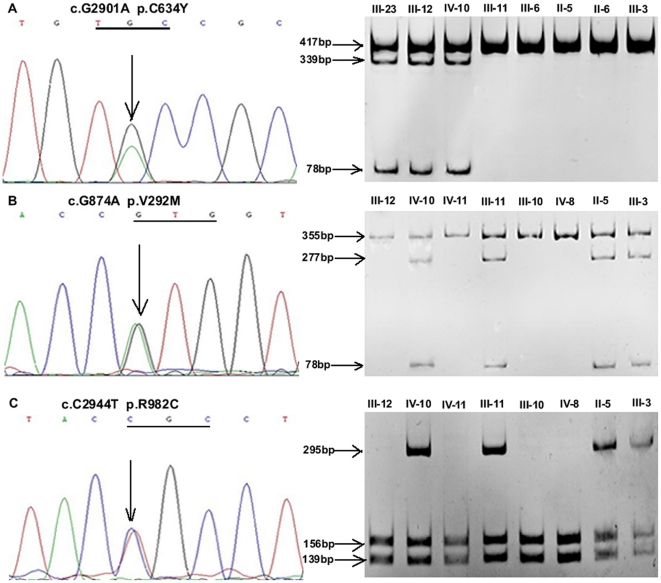
Portion of DNA sequences and polyacrylamide gel electrophoresis analysis of restriction endonuclease digestion. A, Maternal origin MEN 2A (III-23, III-12 and IV-10) were heterozygous for c.G2901A (p.C634Y), whereas paternal origin members (III-11, III-6, II-5, II-6 and III-3) were homozygous for the wild-type allele. This mutation was confirmed by *Kpn* I digestion. B, IV-10 and paternal origin FMTC (III-11, II-5, and III-3) demonstrated a heterozygous c.G874A (p.V292M) mutation which created the restriction enzyme site for *Nco* I, whereas maternal origin members (III-12 and IV-11), and two unaffected paternal members (III-10 and IV-8) showed the normal sequence. C, A heterozygous c.C2944T (p.R982C) polymorphism from IV-10 and paternal origin FMTC (III-11, II-5 and III-3) was found, whereas the maternal origin members III-12 and IV-11 and two unaffected paternal members (III-10 and IV-8) were homozygous for the wild-type allele. This polymorphism was validated by *Rsa* I digestion.

**Figure 3:**
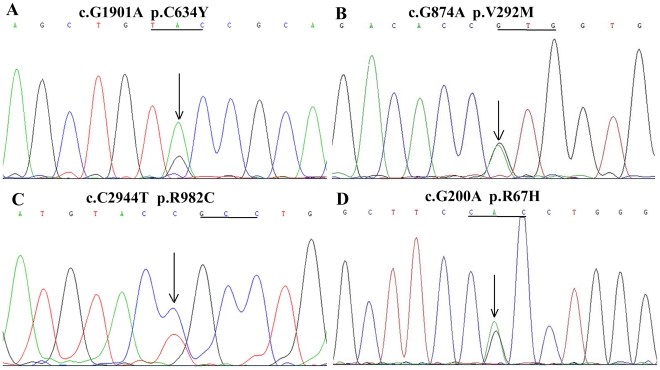
Portion of genomic DNA and RT-PCR product sequencing from tumor tissues. A, Direct sequencing consequences of RT-PCR products from PHEO tissue of III-12 indicated a heterozygous G/A mutation at codon 634, which was consistent with the results of DNA from blood and tumor tissues and whole exome sequencing. B, C, D, Direct sequencing consequences of RT-PCR products from MTC tissue of III-11 indicated heterozygous G/A, C/T, and G/A mutations at codons 292, 982, and 67, respectively, which was consistent with the results of DNA from blood and tumor tissues and whole exome sequencing.

**Figure 4:**
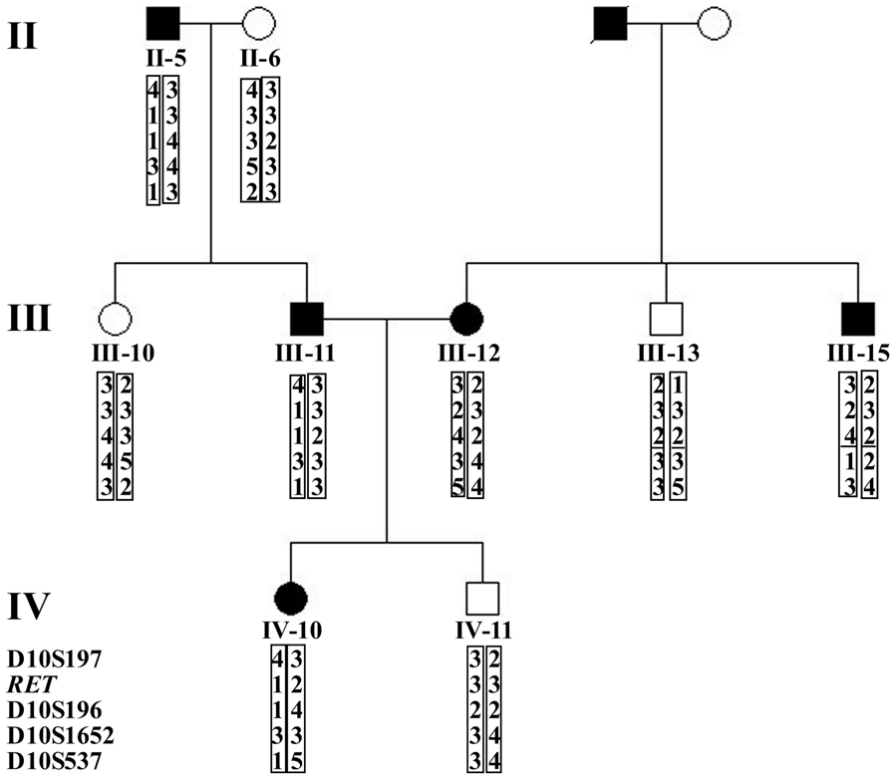
Haplotype analysis. Four microsatellite markers (D10S197, D10S196, D10S1652 and D10S537) from 9 related members (II-5, II-6, III-10, III-11, III-12, III-13, III-15, IV-10 and IV-11) demonstrated that the p.C634Y/V292M/R67H/R982C mutation did not arise on a common chromosome; p.C634Y was of maternal origin and p.V292M/R67H/R982C was paternal and located in a common allele.

The Sanger sequencing scan of the whole *RET* gene was completely consistent with the whole exome sequencing. The proband (III-12) carried the p.C634Y mutation within exon 11, and so did her family members III-15, III-20, III-23 and IV-21. Unfortunately and interestingly, the proband's husband (III-11) and his family members II-5, III-3, III-8, IV-3 and IV-7 carried the p.R67H mutation within exon 2, the p.V292M mutation within exon 5 and the p.R982C polymorphism within exon 18 (Table 1). Thus, their daughter (IV-10) inherited four substitutions of p.C634Y/V292M/R67H/R982C (GenBank accession number for p.C634Y: JF276429; p.V292M/R67H/R982C: JF273638), of which p.C634Y, p.V292M and p.R982C were respectively confirmed by *Kpn* I, *Nco* I and *Rsa* I digestion (Fig. 2). Genomic DNA and RT-PCR product sequencing from tumor tissues were both consistent with the results from blood and presented heterozygosity (Fig. 3). The four alterations were absent in 100 healthy controls. Haplotype analysis of four microsatellite markers from the 9 related members (II-5, II-6, III-10, III-11, III-12, III-13, III-15, IV-10 and IV-11) confirmed that p.C634Y was of maternal origin and p.V292M/R67H/R982C was paternal and located in a common allele (Fig. 4).

## Discussion

We used WES here and successfully confirmed several pathogenic variants in a Chinese family with MEN 2A/FMTC. WES can serve as a genome-wide scan for the causative gene [20]. Moving along the spectrum from rare monogenic disorders to complex common diseases, it is likely that the increasing extent of genetic heterogeneity will need to be matched by increasingly large sample sizes [23]. Meanwhile, although this approach could be underemployed for mutation analysis of known genes, especially those with hot-spot mutations, our case serves as a proper example of genetic diagnosis of a pathogenic gene that harbors a large myriad of exons, particularly those without hot-spot mutational sites, such as Cornelia de Lange syndrome with causative gene *NIPBL* embracing 47 exons without hot-spot mutational sites [24]. WES can reduce the missed detection of some rare mutations in cases with hot-spot mutations and serve as a more direct method than cumbersome Sanger sequencing of a myriad of PCR fragments [19]. Moreover, WES could be a better choice for some diseases with overlapping symptoms that might be ambiguously associated with many pathogenic genes, in which event the detection of mutation by Sanger sequencing could be time-consuming and labor-intensive [18], [19]. The standard sequencing of a single PCR fragment in molecular diagnosis costs 600 Yuan (∼$91) for Zhejiang University-Adinovo Center for Genetic and Genomic Medicine. This price includes consumables, equipment amortization, personnel salary and corporate profit. Therefore, the total cost of sequencing the entire *RET* coding sequence, which requires 21 PCR fragments, is 12600 Yuan (∼$1909). In comparison, the all-inclusive cost of WES per sample is 25000 Yuan (∼$3788), about twice as much as Sanger sequencing. However, WES would be a more cost-effective and robust method for a causative gene embracing more than 42 exons (25200 Yuan, ∼$3819) such as *NIPBL*. Additionally, with rapid commercialization, the cost of WES for gene testing will be considerably reduced in the coming years [19].

The most frequent *RET* mutations responsible for MEN 2A are p.C634Y/R/W/F/S/G/L, which target to one of five cysteine residues in the extracellular domain of *RET* [5], [25]. It seems that different amino acid substitutions within codon 634 present different prevalence in different populations but cause indistinguishable phenotypes, although one study suggested that individuals with p.C634R have significantly more distant metastases at diagnosis than those with p.C634Y/W [25], [26]. In this study, the clinical features seemed similar to those reported by Zhou *et al* [25], which confirmed the correlation of MEN 2A and p.C634Y in Chinese.

Previous studies provided evidence that some variants play roles in modifying other variants in the genesis of MEN 2A or FMTC. The p.V292M mutation maps to the third cadherin-like domain of *RET* and is a gain-of-function alteration with weaker pathogenicity [12]. An Italian MEN 2A patient with p.V292M presented at the age of 44 with classic PHEO and a 4-mm unifocal MTC in the right thyroid lobe [12]. Dvorakova *et al* reported another mutation, p.G321R, within exon 5 in a three-generation Czech kindred with FMTC [27]. Among 4 affected paternal members, one (aged 70) had MTC, one (aged 42) had CCH, and two (aged 27 and 52) had no evidence of either [27]. This indicated that mutations in the extracellular cadherin-like domain cause a comparatively mild clinical phenotype. Clinical investigation in our patients revealed that only one (II-5) had bilateral MTC at the age of 70, one (III-10) had bilateral CCH at age 44, and two (III-8 and III-3) had bilateral multiple nodules in both thyroid lobes at ages 46 and 48, which may be caused by the *RET* p.V292M/R67H/R982C mutation. In contrast to the previous literature, our patients presented with a later onset of MTC or CCH and showed no evidence of PHEO even in the 70-year-old male (II-5), while the previously reported case with p.V292M had PHEO before MTC [12]. The p.V292M/R67H/R982C compound mutation may predispose to the FMTC phenotype. Previously-reported *RET* double mutations associated with MEN 2 are summarized in Table 4. Susceptibility to MEN 2A seems to be impaired by certain *RET* polymorphisms [28]–[30], while polymorphisms involving genes whose products interact with *RET* or components of the *RET* signaling pathway have also been reported to modify the risk of MTC [31], [32]. Iwashita *et al* demonstrated that the transforming activity of *RET* with the *cis* p.V804M/Y806C mutation is dramatically higher than that of *RET* with a single p.V804M or p.Y806C mutation [33], [34]. Kasprzak *et al* indicated that p.V778I might potentially affect the phenotype through an additive effect in the presence of p.V804M in a Spanish family [35]. Recent studies showed that p.Y791F is definitely not a pathogenic mutation for MEN 2, but the p.C634Y/Y791F *cis* double mutation carries a codon 634-like pattern of MTC development and is associated with increased susceptibility to unusually large bilateral PHEO [13], [32]. Meanwhile, Bartsch *et al* concluded that the *RET* p.V804M/R844L *cis* double mutation predisposes the individual to the development of FMTC with a mild phenotype [36]. In these events, it seems that p.Y806C, p.V778I and polymorphism p.Y791F have additive effects to p.V804M and p.C634Y, respectively, while p.R844L has an inhibitory modifying effect on p.V804M. In the present study, entire *RET* screening showed that *cis* p.R67H/R982C combined with p.V292M in *cis* co-segregated with the FMTC phenotype. Since we did not find PHEO, in contrast to the phenotype of p.V292M reported previously, we speculated that the *cis* p.R67H/R982C variant has a modifying effect on the disease and may inhibit the genesis of PHEO, although p.R982C seems to have normal biological and biochemical behavior in *in vitro* expression experiments [14], [37], and p.R67H/R982C was reported to be associated with congenital central hypoventilation syndrome [15].

**Table 4:** Summary of reported *RET* double mutations associated with MEN 2.

Time	Mutation and pattern	Phenotype	Origin	Reference
1999	p.C634R/A640G, *cis*	Calcitonin-producing PHEO	Italy	[43]
2000	p.V804M/Y806C, *cis*	MEN 2B	Japan	[33], [34]
2000	p.V804M/R844L, *cis*	FMTC with a mild phenotype	Germany	[36]
2001	p.V804M/V778I, *cis*	FMTC with corneal nerve thickening	Spain	[35]
2002	p.V804M/S904Y, *cis*	Atypical MEN 2B	Netherlands	[42]
2002	p.C634R/V648I, *trans*	Adrenocorticotropic hormone-producing PHEO	Brazil	[38]
2005	p.C634S/A641S, *cis*	MTC (10/10), PHEO (2/10), HPT (0/10)	Slovakia	[41]
2006	p.V804M/E805K, *cis*	Classical features of MEN 2B	United Kingdom	[31]
2010	p.C634Y/Y791F, *cis*	High penetrance of PHEO	Brazil	[13]

Previous studies have shown that double *RET* mutations may be associated with unusual MEN 2 phenotypes (Table 4). The case with p.C634Y/V292M/R67H/R982C (IV-10) had a more advanced and large bilateral MTC with lymph node metastases (Table 1), in contrast to p.C634Y (including contemporary IV-21) or p.V292M/R67H/R982C (including contemporaries IV-3 and IV-7) carriers, but lacked PHEO and HPT. Nunes *et al* believed that the *RET trans* p.C634R/V648I double mutation may have modified and contributed to the rare MEN 2A phenotype in a patient with bilateral adrenocorticotropin-producing PHEO and MTC [38]. A previous study showed that the potential physiochemical effects of p.V804M and p.E805K substitutions *in silico* are moderately deleterious in isolation but severely deleterious in *cis* combination due to their distinctly synergistic effect [39]. These two cases provided the evidence that these two pathogenic mutations together amount to a more deleterious double mutation than that in isolation, which might explain the higher MTC pathogenicity of p.C634Y/V292M/R67H/R982C due to the additive effect of p.V292M/R67H/R982C and p.C634Y in *trans*. A “second hit” mechanism advocates that allelic imbalance between mutant and wild-type *RET* is a possible mechanism of tumorigenesis in some patients with MEN 2A-related MTC [40]. Our data illustrated that the consistency of sequencing of genomic DNA from blood and tumor tissues and the cDNA from tumor tissues refuted the loss of heterozygosity and are inconsistent with the “second hit” mechanism [40]. However, the wild-type *RET* might play a neutralizing role and thereby partly compensate for the activating effects of the mutant allele, but this protective effect of wild-type *RET* may disappear in the *trans* double mutant, which, from another perspective, could explain the more aggressive pathogenicity of the p.C634Y/V292M/R67H/R982C compound mutation [40]. Due to the young age of 22 years in IV-10, the combination of p.C634Y and p.V292M/R67H/R982C, and the reduced penetrance of PHEO and HPT, it is too early to ascertain whether she will develop PHEO and HPT.

Briefly, WES provides an instructive, comprehensive, fast and accurate method for genetic diagnosis of a disease whose pathogenic genes embrace a large cohort of exons. Our case suggests the importance of extending *RET* sequencing studies to the gene's entire coding region, even though initial testing of a patient with clinical signs of MEN-2 reveals *RET* hot-spot mutations, or when genotype-phenotype discrepancies emerge. Meanwhile, in the present study, the p.R67H/R982C polymorphism had a modifying effect on the disease and may inhibit the genesis of PHEO, and p.C634Y/V292M/R67H/R982C may lead to higher MTC pathogenicity due to the additive effect of p.V292M/R67H/R982C and p.C634Y in *trans* combination.
